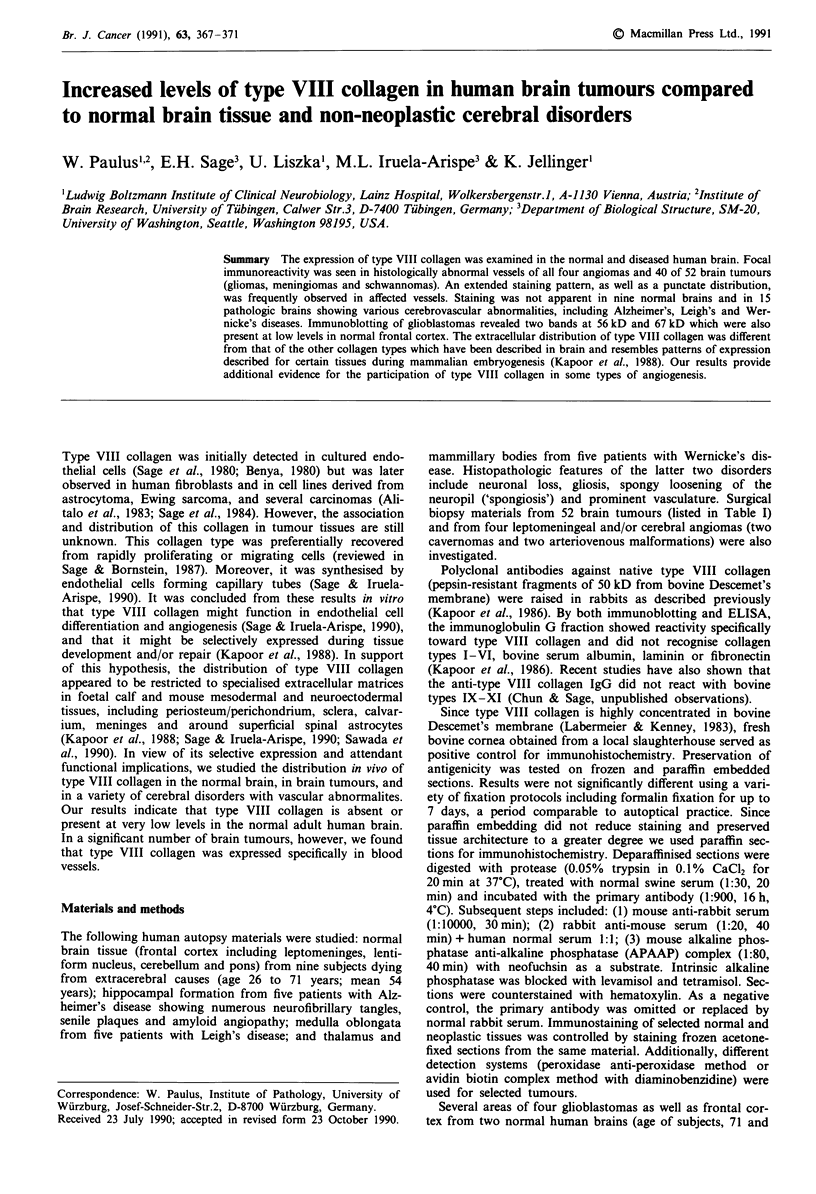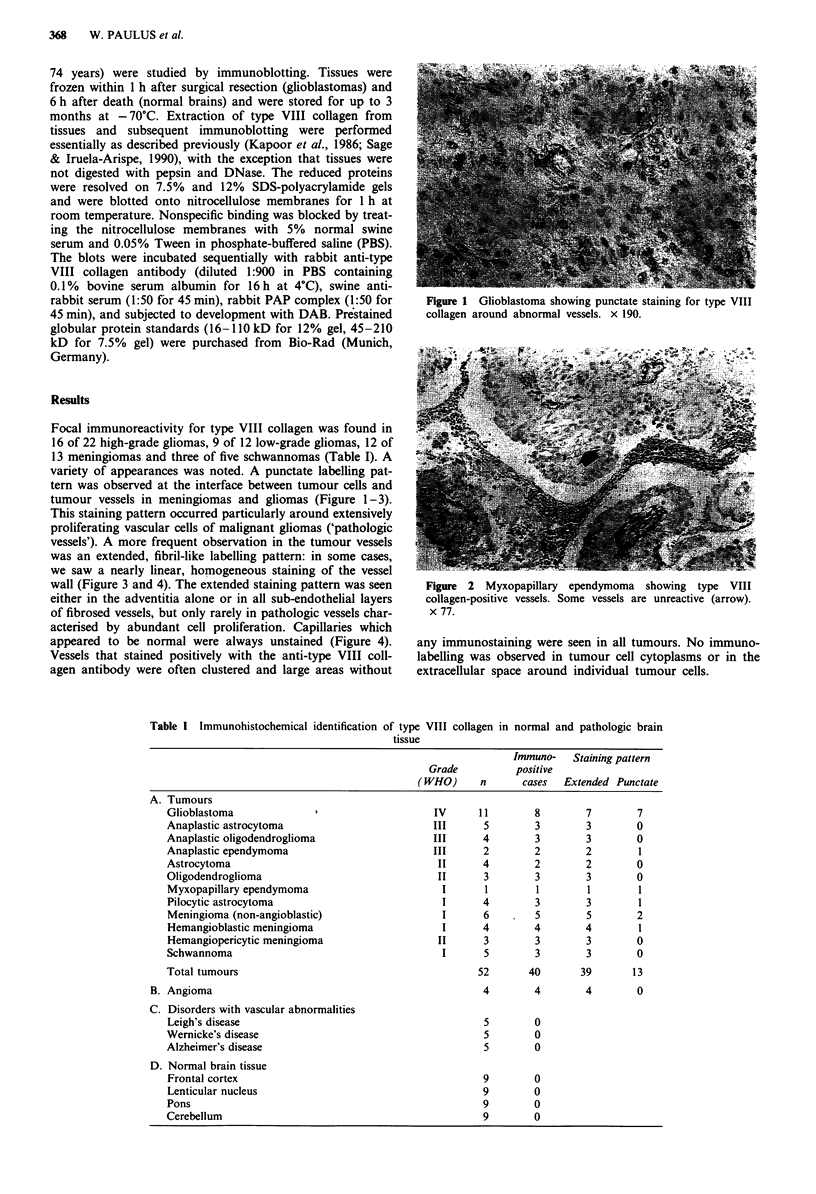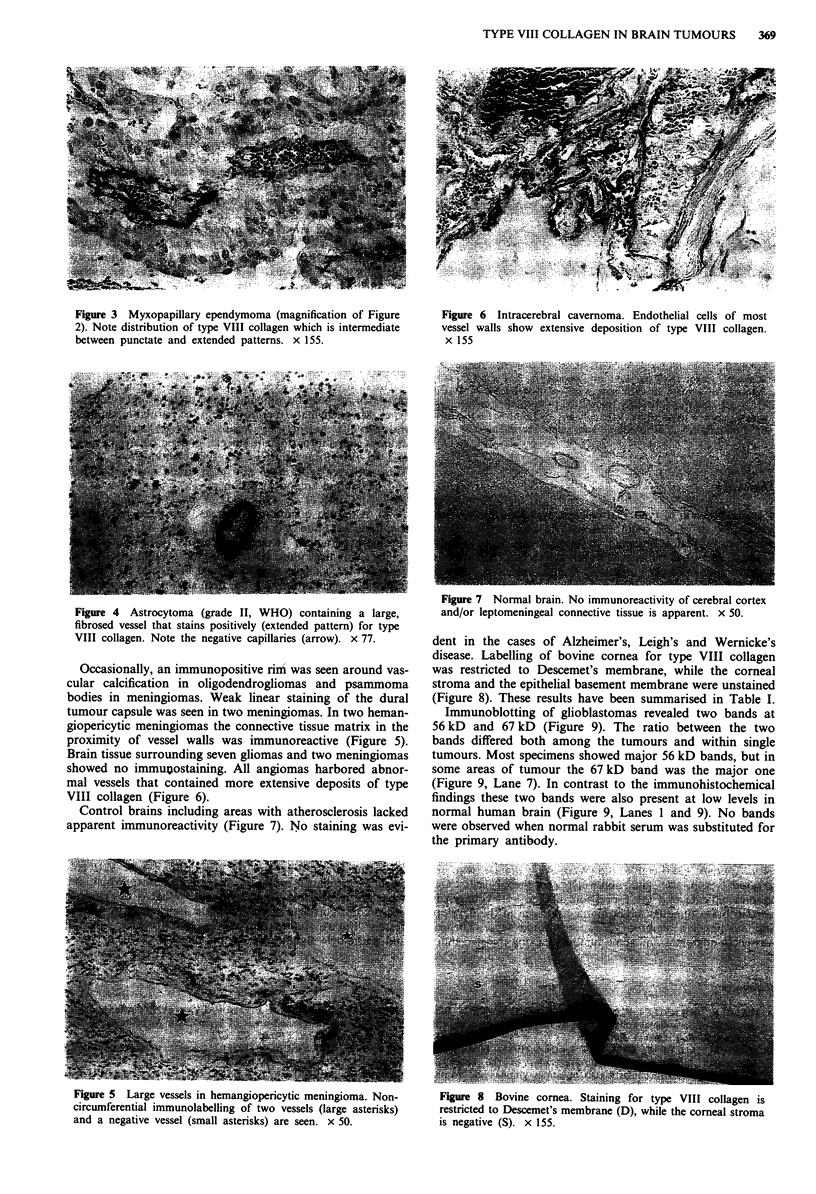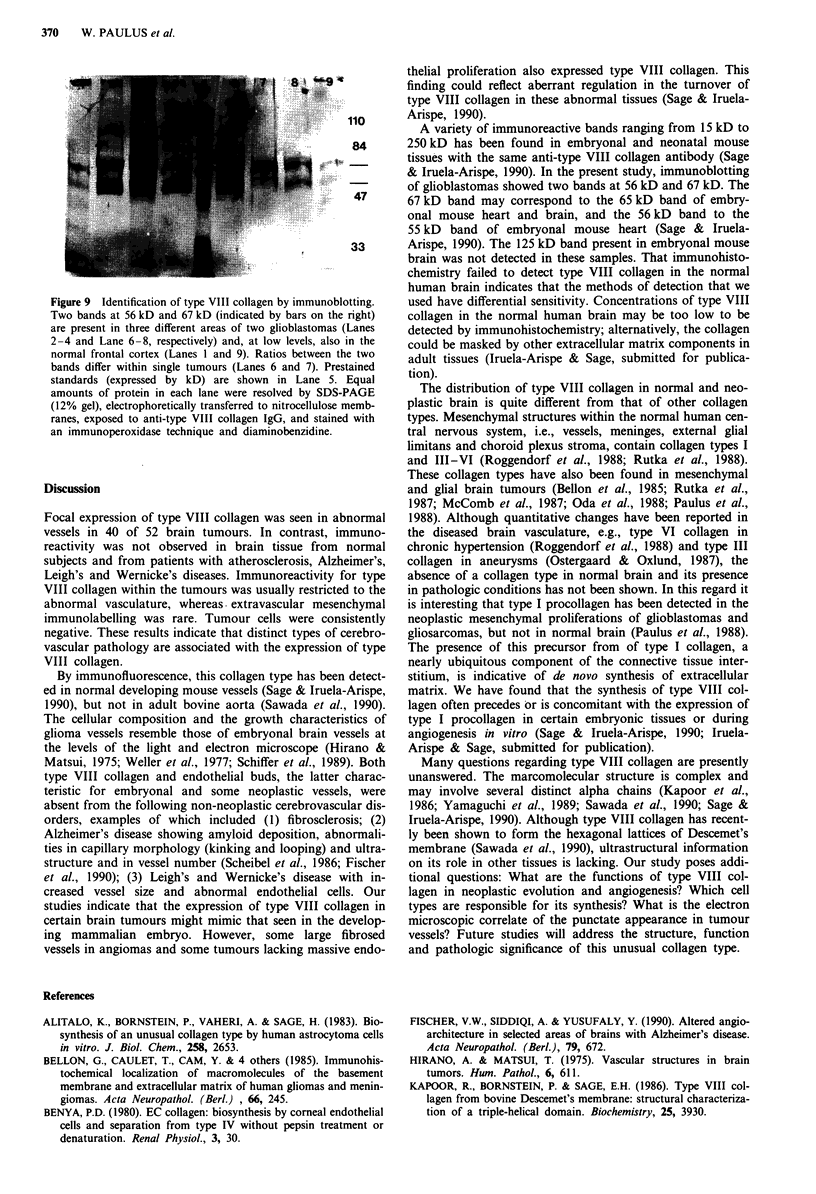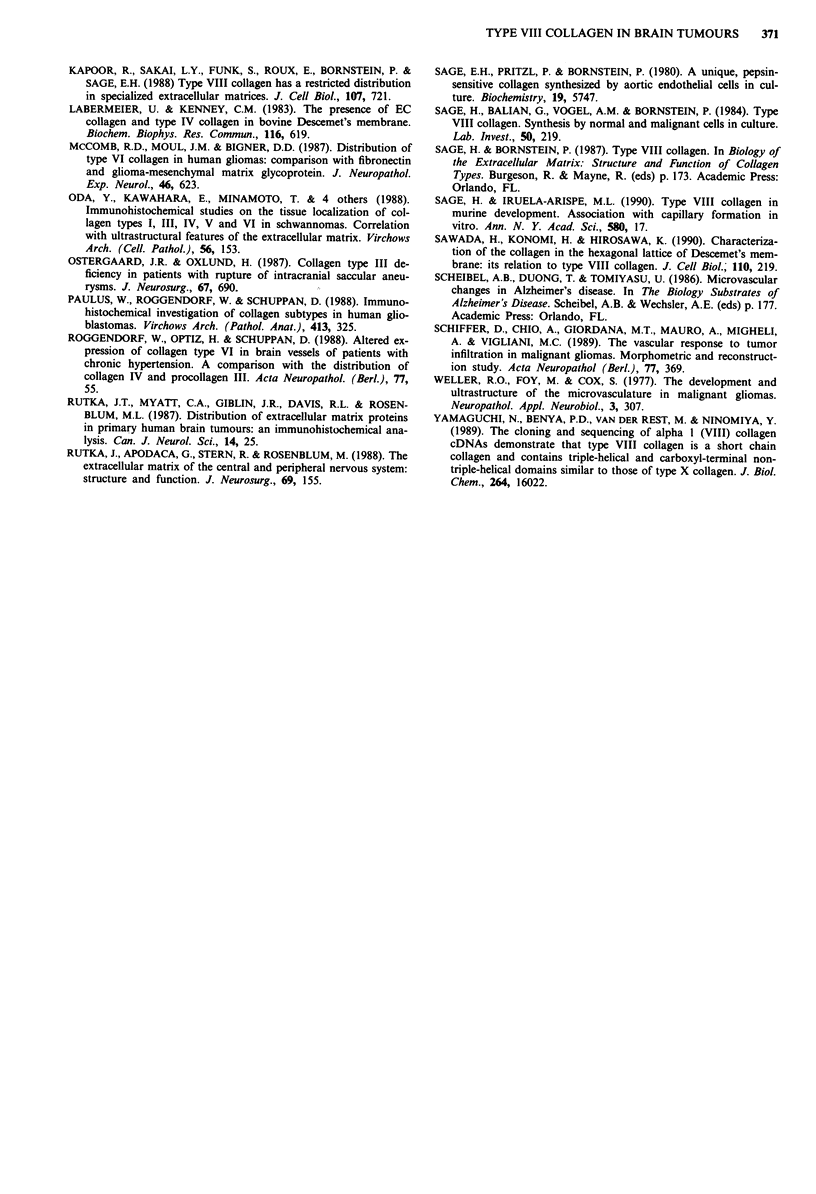# Increased levels of type VIII collagen in human brain tumours compared to normal brain tissue and non-neoplastic cerebral disorders.

**DOI:** 10.1038/bjc.1991.87

**Published:** 1991-03

**Authors:** W. Paulus, E. H. Sage, U. Liszka, M. L. Iruela-Arispe, K. Jellinger

**Affiliations:** Ludwig Boltzmann Institute of Clinical Neurobiology, Lainz Hospital, Vienna, Austria.

## Abstract

**Images:**


Br. J. Cancer (1991), 63, 367-371

© Macmillan Press Ltd., 1991

### Increased levels of type VIII collagen in human brain tumours compared to normal brain tissue and non-neoplastic cerebral disorders

W. Paulus[1,2], E.H. Sage[3], U. Liszka[1], M.L. Iruela-Arispe[3] & K. Jellinger[1]

[1]*Ludwig Boltzmann Institute of Clinical Neurobiology, Lainz Hospital, Wolkersbergenstr.1, A-1130 Vienna, Austria;* [2]*Institute of Brain Research, University of Tübingen, Calwer Str.3, D-7400 Tübingen, Germany;* [3]*Department of Biological Structure, SM-20, University of Washington, Seattle, Washington 98195, USA.*

**Summary** The expression of type VIII collagen was examined in the normal and diseased human brain. Focal immunoreactivity was seen in histologically abnormal vessels of all four angiomas and 40 of 52 brain tumours (gliomas, meningiomas and schwannomas). An extended staining pattern, as well as a punctate distribution, was frequently observed in affected vessels. Staining was not apparent in nine normal brains and in 15 pathologic brains showing various cerebrovascular abnormalities, including Alzheimer's, Leigh's and Wernicke's diseases. Immunoblotting of glioblastomas revealed two bands at 56 kD and 67 kD which were also present at low levels in normal frontal cortex. The extracellular distribution of type VIII collagen was different from that of the other collagen types which have been described in brain and resembles patterns of expression described for certain tissues during mammalian embryogenesis (Kapoor *et al.*, 1988). Our results provide additional evidence for the participation of type VIII collagen in some types of angiogenesis.

Type VIII collagen was initially detected in cultured endothelial cells (Sage *et al.*, 1980; Benya, 1980) but was later observed in human fibroblasts and in cell lines derived from astrocytoma, Ewing sarcoma, and several carcinomas (Alitalo *et al.*, 1983; Sage *et al.*, 1984). However, the association and distribution of this collagen in tumour tissues are still unknown. This collagen type was preferentially recovered from rapidly proliferating or migrating cells (reviewed in Sage & Bornstein, 1987). Moreover, it was synthesised by endothelial cells forming capillary tubes (Sage & Iruela-Arispe, 1990). It was concluded from these results *in vitro* that type VIII collagen might function in endothelial cell differentiation and angiogenesis (Sage & Iruela-Arispe, 1990), and that it might be selectively expressed during tissue development and/or repair (Kapoor *et al.*, 1988). In support of this hypothesis, the distribution of type VIII collagen appeared to be restricted to specialised extracellular matrices in foetal calf and mouse mesodermal and neuroectodermal tissues, including periosteum/perichondrium, sclera, calvarium, meninges and around superficial spinal astrocytes (Kapoor *et al.*, 1988; Sage & Iruela-Arispe, 1990; Sawada *et al.*, 1990). In view of its selective expression and attendant functional implications, we studied the distribution *in vivo* of type VIII collagen in the normal brain, in brain tumours, and in a variety of cerebral disorders with vascular abnormalites. Our results indicate that type VIII collagen is absent or present at very low levels in the normal adult human brain. In a significant number of brain tumours, however, we found that type VIII collagen was expressed specifically in blood vessels.

### Materials and methods

The following human autopsy materials were studied: normal brain tissue (frontal cortex including leptomeninges, lentiform nucleus, cerebellum and pons) from nine subjects dying from extracerebral causes (age 26 to 71 years; mean 54 years); hippocampal formation from five patients with Alzheimer's disease showing numerous neurofibrillary tangles, senile plaques and amyloid angiopathy; medulla oblongata from five patients with Leigh's disease; and thalamus and mammillary bodies from five patients with Wernicke's disease. Histopathologic features of the latter two disorders include neuronal loss, gliosis, spongy loosening of the neuropil ('spongiosis') and prominent vasculature. Surgical biopsy materials from 52 brain tumours (listed in Table I) and from four leptomeningeal and/or cerebral angiomas (two cavernomas and two arteriovenous malformations) were also investigated.

Polyclonal antibodies against native type VIII collagen (pepsin-resistant fragments of 50 kD from bovine Descemet's membrane) were raised in rabbits as described previously (Kapoor *et al.*, 1986). By both immunoblotting and ELISA, the immunoglobulin G fraction showed reactivity specifically toward type VIII collagen and did not recognise collagen types I-VI, bovine serum albumin, laminin or fibronectin (Kapoor *et al.*, 1986). Recent studies have also shown that the anti-type VIII collagen IgG did not react with bovine types IX-XI (Chun & Sage, unpublished observations).

Since type VIII collagen is highly concentrated in bovine Descemet's membrane (Labermeier & Kenney, 1983), fresh bovine cornea obtained from a local slaughterhouse served as positive control for immunohistochemistry. Preservation of antigenicity was tested on frozen and paraffin embedded sections. Results were not significantly different using a variety of fixation protocols including formalin fixation for up to 7 days, a period comparable to autoptical practice. Since paraffin embedding did not reduce staining and preserved tissue architecture to a greater degree we used paraffin sections for immunohistochemistry. Deparaffinised sections were digested with protease (0.05% trypsin in 0.1% $CaCl_2$ for 20 min at 37°C), treated with normal swine serum (1:30, 20 min) and incubated with the primary antibody (1:900, 16 h, 4°C). Subsequent steps included: (1) mouse anti-rabbit serum (1:10000, 30 min); (2) rabbit anti-mouse serum (1:20, 40 min) + human normal serum 1:1; (3) mouse alkaline phosphatase anti-alkaline phosphatase (APAAP) complex (1:80, 40 min) with neofuchsin as a substrate. Intrinsic alkaline phosphatase was blocked with levamisol and tetramisol. Sections were counterstained with hematoxylin. As a negative control, the primary antibody was omitted or replaced by normal rabbit serum. Immunostaining of selected normal and neoplastic tissues was controlled by staining frozen acetone-fixed sections from the same material. Additionally, different detection systems (peroxidase anti-peroxidase method or avidin biotin complex method with diaminobenzidine) were used for selected tumours.

Several areas of four glioblastomas as well as frontal cortex from two normal human brains (age of subjects, 71 and

Correspondence: W. Paulus, Institute of Pathology, University of Würzburg, Josef-Schneider-Str.2, D-8700 Würzburg, Germany.
Received 23 July 1990; accepted in revised form 23 October 1990.

74 years) were studied by immunoblotting. Tissues were frozen within 1 h after surgical resection (glioblastomas) and 6 h after death (normal brains) and were stored for up to 3 months at −70°C. Extraction of type VIII collagen from tissues and subsequent immunoblotting were performed essentially as described previously (Kapoor *et al.*, 1986; Sage & Iruela-Arispe, 1990), with the exception that tissues were not digested with pepsin and DNase. The reduced proteins were resolved on 7.5% and 12% SDS-polyacrylamide gels and were blotted onto nitrocellulose membranes for 1 h at room temperature. Nonspecific binding was blocked by treating the nitrocellulose membranes with 5% normal swine serum and 0.05% Tween in phosphate-buffered saline (PBS). The blots were incubated sequentially with rabbit anti-type VIII collagen antibody (diluted 1:900 in PBS containing 0.1% bovine serum albumin for 16 h at 4°C), swine anti-rabbit serum (1:50 for 45 min), rabbit PAP complex (1:50 for 45 min), and subjected to development with DAB. Prestained globular protein standards (16–110 kD for 12% gel, 45–210 kD for 7.5% gel) were purchased from Bio-Rad (Munich, Germany).

### Results

Focal immunoreactivity for type VIII collagen was found in 16 of 22 high-grade gliomas, 9 of 12 low-grade gliomas, 12 of 13 meningiomas and three of five schwannomas (Table I). A variety of appearances was noted. A punctate labelling pattern was observed at the interface between tumour cells and tumour vessels in meningiomas and gliomas (Figure 1–3). This staining pattern occurred particularly around extensively proliferating vascular cells of malignant gliomas ('pathologic vessels'). A more frequent observation in the tumour vessels was an extended, fibril-like labelling pattern: in some cases, we saw a nearly linear, homogeneous staining of the vessel wall (Figure 3 and 4). The extended staining pattern was seen either in the adventitia alone or in all sub-endothelial layers of fibrosed vessels, but only rarely in pathologic vessels characterised by abundant cell proliferation. Capillaries which appeared to be normal were always unstained (Figure 4). Vessels that stained positively with the anti-type VIII collagen antibody were often clustered and large areas without any immunostaining were seen in all tumours. No immunolabelling was observed in tumour cell cytoplasms or in the extracellular space around individual tumour cells.

**Figure 1** Glioblastoma showing punctate staining for type VIII collagen around abnormal vessels. × 190.

**Figure 2** Myxopapillary ependymoma showing type VIII collagen-positive vessels. Some vessels are unreactive (arrow). × 77.

**Table I** Immunohistochemical identification of type VIII collagen in normal and pathologic brain tissue

| | *Grade (WHO)* | *n* | *Immuno-positive cases* | *Staining pattern Extended* | *Staining pattern Punctate* |
|---|---|---|---|---|---|
| A. Tumours | | | | | |
| Glioblastoma | IV | 11 | 8 | 7 | 7 |
| Anaplastic astrocytoma | III | 5 | 3 | 3 | 0 |
| Anaplastic oligodendroglioma | III | 4 | 3 | 3 | 0 |
| Anaplastic ependymoma | III | 2 | 2 | 2 | 1 |
| Astrocytoma | II | 4 | 2 | 2 | 0 |
| Oligodendroglioma | II | 3 | 3 | 3 | 0 |
| Myxopapillary ependymoma | I | 1 | 1 | 1 | 1 |
| Pilocytic astrocytoma | I | 4 | 3 | 3 | 1 |
| Meningioma (non-angioblastic) | I | 6 | 5 | 5 | 2 |
| Hemangioblastic meningioma | I | 4 | 4 | 4 | 1 |
| Hemangiopericytic meningioma | II | 3 | 3 | 3 | 0 |
| Schwannoma | I | 5 | 3 | 3 | 0 |
| Total tumours | | 52 | 40 | 39 | 13 |
| B. Angioma | | 4 | 4 | 4 | 0 |
| C. Disorders with vascular abnormalities | | | | | |
| Leigh's disease | | 5 | 0 | | |
| Wernicke's disease | | 5 | 0 | | |
| Alzheimer's disease | | 5 | 0 | | |
| D. Normal brain tissue | | | | | |
| Frontal cortex | | 9 | 0 | | |
| Lenticular nucleus | | 9 | 0 | | |
| Pons | | 9 | 0 | | |
| Cerebellum | | 9 | 0 | | |

**Figure 3** Myxopapillary ependymoma (magnification of Figure 2). Note distribution of type VIII collagen which is intermediate between punctate and extended patterns. × 155.

**Figure 4** Astrocytoma (grade II, WHO) containing a large, fibrosed vessel that stains positively (extended pattern) for type VIII collagen. Note the negative capillaries (arrow). × 77.

Occasionally, an immunopositive rim was seen around vascular calcification in oligodendrogliomas and psammoma bodies in meningiomas. Weak linear staining of the dural tumour capsule was seen in two meningiomas. In two hemangiopericytic meningiomas the connective tissue matrix in the proximity of vessel walls was immunoreactive (Figure 5). Brain tissue surrounding seven gliomas and two meningiomas showed no immunostaining. All angiomas harbored abnormal vessels that contained more extensive deposits of type VIII collagen (Figure 6).

Control brains including areas with atherosclerosis lacked apparent immunoreactivity (Figure 7). No staining was evident in the cases of Alzheimer's, Leigh's and Wernicke's disease. Labelling of bovine cornea for type VIII collagen was restricted to Descemet's membrane, while the corneal stroma and the epithelial basement membrane were unstained (Figure 8). These results have been summarised in Table I.

Immunoblotting of glioblastomas revealed two bands at 56 kD and 67 kD (Figure 9). The ratio between the two bands differed both among the tumours and within single tumours. Most specimens showed major 56 kD bands, but in some areas of tumour the 67 kD band was the major one (Figure 9, Lane 7). In contrast to the immunohistochemical findings these two bands were also present at low levels in normal human brain (Figure 9, Lanes 1 and 9). No bands were observed when normal rabbit serum was substituted for the primary antibody.

**Figure 5** Large vessels in hemangiopericytic meningioma. Non-circumferential immunolabelling of two vessels (large asterisks) and a negative vessel (small asterisks) are seen. × 50.

**Figure 6** Intracerebral cavernoma. Endothelial cells of most vessel walls show extensive deposition of type VIII collagen. × 155

**Figure 7** Normal brain. No immunoreactivity of cerebral cortex and/or leptomeningeal connective tissue is apparent. × 50.

**Figure 8** Bovine cornea. Staining for type VIII collagen is restricted to Descemet's membrane (D), while the corneal stroma is negative (S). × 155.

**Figure 9** Identification of type VIII collagen by immunoblotting. Two bands at 56 kD and 67 kD (indicated by bars on the right) are present in three different areas of two glioblastomas (Lanes 2–4 and Lane 6–8, respectively) and, at low levels, also in the normal frontal cortex (Lanes 1 and 9). Ratios between the two bands differ within single tumours (Lanes 6 and 7). Prestained standards (expressed by kD) are shown in Lane 5. Equal amounts of protein in each lane were resolved by SDS-PAGE (12% gel), electrophoretically transferred to nitrocellulose membranes, exposed to anti-type VIII collagen IgG, and stained with an immunoperoxidase technique and diaminobenzidine.

### Discussion

Focal expression of type VIII collagen was seen in abnormal vessels in 40 of 52 brain tumours. In contrast, immunoreactivity was not observed in brain tissue from normal subjects and from patients with atherosclerosis, Alzheimer's, Leigh's and Wernicke's diseases. Immunoreactivity for type VIII collagen within the tumours was usually restricted to the abnormal vasculature, whereas extravascular mesenchymal immunolabelling was rare. Tumour cells were consistently negative. These results indicate that distinct types of cerebrovascular pathology are associated with the expression of type VIII collagen.

By immunofluorescence, this collagen type has been detected in normal developing mouse vessels (Sage & Iruela-Arispe, 1990), but not in adult bovine aorta (Sawada *et al.*, 1990). The cellular composition and the growth characteristics of glioma vessels resemble those of embryonal brain vessels at the levels of the light and electron microscope (Hirano & Matsui, 1975; Weller *et al.*, 1977; Schiffer *et al.*, 1989). Both type VIII collagen and endothelial buds, the latter characteristic for embryonal and some neoplastic vessels, were absent from the following non-neoplastic cerebrovascular disorders, examples of which included (1) fibrosclerosis; (2) Alzheimer's disease showing amyloid deposition, abnormalities in capillary morphology (kinking and looping) and ultrastructure and in vessel number (Scheibel *et al.*, 1986; Fischer *et al.*, 1990); (3) Leigh's and Wernicke's disease with increased vessel size and abnormal endothelial cells. Our studies indicate that the expression of type VIII collagen in certain brain tumours might mimic that seen in the developing mammalian embryo. However, some large fibrosed vessels in angiomas and some tumours lacking massive endothelial proliferation also expressed type VIII collagen. This finding could reflect aberrant regulation in the turnover of type VIII collagen in these abnormal tissues (Sage & Iruela-Arispe, 1990).

A variety of immunoreactive bands ranging from 15 kD to 250 kD has been found in embryonal and neonatal mouse tissues with the same anti-type VIII collagen antibody (Sage & Iruela-Arispe, 1990). In the present study, immunoblotting of glioblastomas showed two bands at 56 kD and 67 kD. The 67 kD band may correspond to the 65 kD band of embryonal mouse heart and brain, and the 56 kD band to the 55 kD band of embryonal mouse heart (Sage & Iruela-Arispe, 1990). The 125 kD band present in embryonal mouse brain was not detected in these samples. That immunohistochemistry failed to detect type VIII collagen in the normal human brain indicates that the methods of detection that we used have differential sensitivity. Concentrations of type VIII collagen in the normal human brain may be too low to be detected by immunohistochemistry; alternatively, the collagen could be masked by other extracellular matrix components in adult tissues (Iruela-Arispe & Sage, submitted for publication).

The distribution of type VIII collagen in normal and neoplastic brain is quite different from that of other collagen types. Mesenchymal structures within the normal human central nervous system, i.e., vessels, meninges, external glial limitans and choroid plexus stroma, contain collagen types I and III–VI (Roggendorf *et al.*, 1988; Rutka *et al.*, 1988). These collagen types have also been found in mesenchymal and glial brain tumours (Bellon *et al.*, 1985; Rutka *et al.*, 1987; McComb *et al.*, 1987; Oda *et al.*, 1988; Paulus *et al.*, 1988). Although quantitative changes have been reported in the diseased brain vasculature, e.g., type VI collagen in chronic hypertension (Roggendorf *et al.*, 1988) and type III collagen in aneurysms (Ostergaard & Oxlund, 1987), the absence of a collagen type in normal brain and its presence in pathologic conditions has not been shown. In this regard it is interesting that type I procollagen has been detected in the neoplastic mesenchymal proliferations of glioblastomas and gliosarcomas, but not in normal brain (Paulus *et al.*, 1988). The presence of this precursor from of type I collagen, a nearly ubiquitous component of the connective tissue interstitium, is indicative of *de novo* synthesis of extracellular matrix. We have found that the synthesis of type VIII collagen often precedes or is concomitant with the expression of type I procollagen in certain embryonic tissues or during angiogenesis *in vitro* (Sage & Iruela-Arispe, 1990; Iruela-Arispe & Sage, submitted for publication).

Many questions regarding type VIII collagen are presently unanswered. The marcomolecular structure is complex and may involve several distinct alpha chains (Kapoor *et al.*, 1986; Yamaguchi *et al.*, 1989; Sawada *et al.*, 1990; Sage & Iruela-Arispe, 1990). Although type VIII collagen has recently been shown to form the hexagonal lattices of Descemet's membrane (Sawada *et al.*, 1990), ultrastructural information on its role in other tissues is lacking. Our study poses additional questions: What are the functions of type VIII collagen in neoplastic evolution and angiogenesis? Which cell types are responsible for its synthesis? What is the electron microscopic correlate of the punctate appearance in tumour vessels? Future studies will address the structure, function and pathologic significance of this unusual collagen type.

### References

ALITALO, K., BORNSTEIN, P., VAHERI, A. & SAGE, H. (1983). Biosynthesis of an unusual collagen type by human astrocytoma cells *in vitro*. *J. Biol. Chem.*, **258**, 2653.

BELLON, G., CAULET, T., CAM, Y. & 4 others (1985). Immunohistochemical localization of macromolecules of the basement membrane and extracellular matrix of human gliomas and meningiomas. *Acta Neuropathol. (Berl.)* , **66**, 245.

BENYA, P.D. (1980). EC collagen: biosynthesis by corneal endothelial cells and separation from type IV without pepsin treatment or denaturation. *Renal Physiol.*, **3**, 30.

FISCHER, V.W., SIDDIQI, A. & YUSUFALY, Y. (1990). Altered angioarchitecture in selected areas of brains with Alzheimer's disease. *Acta Neuropathol. (Berl.)*, **79**, 672.

HIRANO, A. & MATSUI, T. (1975). Vascular structures in brain tumors. *Hum. Pathol.*, **6**, 611.

KAPOOR, R., BORNSTEIN, P. & SAGE, E.H. (1986). Type VIII collagen from bovine Descemet's membrane: structural characterization of a triple-helical domain. *Biochemistry*, **25**, 3930.

KAPOOR, R., SAKAI, L.Y., FUNK, S., ROUX, E., BORNSTEIN, P. & SAGE, E.H. (1988) Type VIII collagen has a restricted distribution in specialized extracellular matrices. *J. Cell Biol.*, **107**, 721.

LABERMEIER, U. & KENNEY, C.M. (1983). The presence of EC collagen and type IV collagen in bovine Descemet's membrane. *Biochem. Biophys. Res. Commun.*, **116**, 619.

MCCOMB, R.D., MOUL, J.M. & BIGNER, D.D. (1987). Distribution of type VI collagen in human gliomas: comparison with fibronectin and glioma-mesenchymal matrix glycoprotein. *J. Neuropathol. Exp. Neurol.*, **46**, 623.

ODA, Y., KAWAHARA, E., MINAMOTO, T. & 4 others (1988). Immunohistochemical studies on the tissue localization of collagen types I, III, IV, V and VI in schwannomas. Correlation with ultrastructural features of the extracellular matrix. *Virchows Arch. (Cell. Pathol.)*, **56**, 153.

OSTERGAARD, J.R. & OXLUND, H. (1987). Collagen type III deficiency in patients with rupture of intracranial saccular aneurysms. *J. Neurosurg.*, **67**, 690.

PAULUS, W., ROGGENDORF, W. & SCHUPPAN, D. (1988). Immunohistochemical investigation of collagen subtypes in human glioblastomas. *Virchows Arch. (Pathol. Anat.)*, **413**, 325.

ROGGENDORF, W., OPTIZ, H. & SCHUPPAN, D. (1988). Altered expression of collagen type VI in brain vessels of patients with chronic hypertension. A comparison with the distribution of collagen IV and procollagen III. *Acta Neuropathol. (Berl.)*, **77**, 55.

RUTKA, J.T., MYATT, C.A., GIBLIN, J.R., DAVIS, R.L. & ROSENBLUM, M.L. (1987). Distribution of extracellular matrix proteins in primary human brain tumours: an immunohistochemical analysis. *Can. J. Neurol. Sci.*, **14**, 25.

RUTKA, J., APODACA, G., STERN, R. & ROSENBLUM, M. (1988). The extracellular matrix of the central and peripheral nervous system: structure and function. *J. Neurosurg.*, **69**, 155.

SAGE, E.H., PRITZL, P. & BORNSTEIN, P. (1980). A unique, pepsin-sensitive collagen synthesized by aortic endothelial cells in culture. *Biochemistry*, **19**, 5747.

SAGE, H., BALIAN, G., VOGEL, A.M. & BORNSTEIN, P. (1984). Type VIII collagen. Synthesis by normal and malignant cells in culture. *Lab. Invest.*, **50**, 219.

SAGE, H. & BORNSTEIN, P. (1987). Type VIII collagen. In *Biology of the Extracellular Matrix: Structure and Function of Collagen Types*. Burgeson, R. & Mayne, R. (eds) p. 173. Academic Press: Orlando, FL.

SAGE, H. & IRUELA-ARISPE, M.L. (1990). Type VIII collagen in murine development. Association with capillary formation in vitro. *Ann. N. Y. Acad. Sci.*, **580**, 17.

SAWADA, H., KONOMI, H. & HIROSAWA, K. (1990). Characterization of the collagen in the hexagonal lattice of Descemet's membrane: its relation to type VIII collagen. *J. Cell Biol.*, **110**, 219.

SCHEIBEL, A.B., DUONG, T. & TOMIYASU, U. (1986). Microvascular changes in Alzheimer's disease. In *The Biology Substrates of Alzheimer's Disease*. Scheibel, A.B. & Wechsler, A.E. (eds) p. 177. Academic Press: Orlando, FL.

SCHIFFER, D., CHIO, A., GIORDANA, M.T., MAURO, A., MIGHELI, A. & VIGLIANI, M.C. (1989). The vascular response to tumor infiltration in malignant gliomas. Morphometric and reconstruction study. *Acta Neuropathol (Berl.)*, **77**, 369.

WELLER, R.O., FOY, M. & COX, S. (1977). The development and ultrastructure of the microvasculature in malignant gliomas. *Neuropathol. Appl. Neurobiol.*, **3**, 307.

YAMAGUCHI, N., BENYA, P.D., VAN DER REST, M. & NINOMIYA, Y. (1989). The cloning and sequencing of alpha 1 (VIII) collagen cDNAs demonstrate that type VIII collagen is a short chain collagen and contains triple-helical and carboxyl-terminal non-triple-helical domains similar to those of type X collagen. *J. Biol. Chem.*, **264**, 16022.